# Effects of microbial inoculants, cellulase, and sodium diacetate on fermentation profiles, bacterial succession, and predicted functional profiles of alfalfa silage

**DOI:** 10.1186/s40643-026-01104-6

**Published:** 2026-08-19

**Authors:** Yanfen Li, Lizhuang Wu, Xaysana Panyavong, Jonggeun Kim

**Affiliations:** 1 Graduate School of International Agricultural Technology, Seoul National University, 1447, Pyeongchang-Daero, Daehwa, PyeongChang, Gangwon 25354 South Korea; 2 Institute of Green-Bio Science Technology, Research Institute of Eco-Friendly Livestock Science, Seoul National University, PyeongChang, 25354 South Korea

**Keywords:** Microbial inoculants, Cellulase, Sodium diacetate, Microbial community, Predicted metabolic pathway

## Abstract

Alfalfa is a high-protein forage crop widely used in ruminant production, yet its ensiling is generally challenged by low water-soluble carbohydrate content and high buffering capacity. This study systematically evaluated the effects of microbial inoculants, cellulase, and sodium diacetate on fermentation quality, microbial succession, and predicted metabolic functions of alfalfa silage using fermentation analyses and 16S rRNA sequencing. Lactic acid was detected only in the microbial inoculant-treated silages on day 1, accompanied by a rapid decline in pH below 5.8 (*p* < 0.05). After 60 days, all microbial inoculants except BSM lowered pH below 4.5 and reduced ammonia nitrogen (NH₃-N) (*p* < 0.05), while promoting *Lactiplantibacillus*- or *Pediococcus*-dominated communities and suppressing *Enterobacter* and *Serratia* (*p* < 0.05). Predicted functional profiles suggested lower lysine degradation and enhanced lysine biosynthesis. Among inoculants, KL yielded the most favorable profile, with the highest *Lactiplantibacillus* abundance (78.23%), lower gas loss, Chao1, Shannon, and NH₃-N (4.35% total nitrogen (TN)). Cellulase improved fermentation quality compared with the control and displayed the lowest fiber content, but showed slower early acidification, resulting in higher NH₃-N (6.46% TN), gas loss, and *Enterobacter* abundance (2.94%) after 60 days (*p* < 0.05). Sodium diacetate followed a distinct pathway, producing acetic acid (1.32% dry matter (DM)) rather than lactic acid on day 1 and reducing pH to 5.58. After 60 days, sodium diacetate produced well-preserved silage with a lower pH (4.28), NH₃-N (3.72% TN), and gas loss, higher lactic acid concentration (6.93% DM) (*p* < 0.05), and *Lactiplantibacillus*-dominated bacterial community. These findings highlight that silage additives govern fermentation outcomes through distinct mechanisms, with the KL inoculant and sodium diacetate providing the most effective preservation strategies in this study.

**Graphical abstract:**

**Figure:**
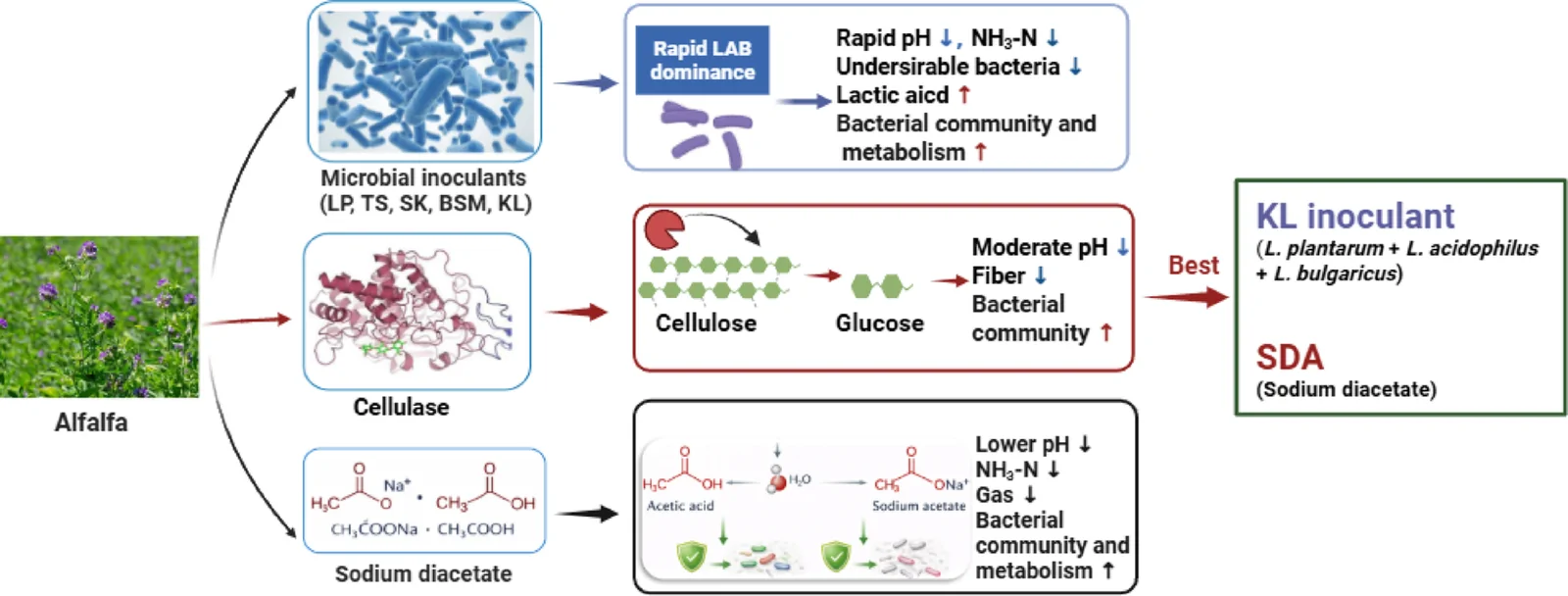

## Introduction

In South Korea, although the forage self-sufficiency rate has reached approximately 78%, rice straw accounts for an excessively high proportion of forage supply, while high-quality roughage accounts for only about 30% forage (MAFRA 2024). Meanwhile, the demand for high-quality forage resources has continued to increase in livestock industry, especially alfalfa. Alfalfa (*Medicago sativa* L.), a perennial legume belonging to the family Fabaceae, is widely recognized as a high-quality forage crop. Alfalfa is one of major imported forage types in South Korea, accounting for approximately 16% of imported forage (MAFRA 2024). Kim et al. (2023) speculated that demand for alfalfa will grow at an average annual rate of 2.3% in South Korea. Therefore, it is important to improve the preservation and utilization efficiency of alfalfa for enhancing high-quality forage supply and reducing dependence on imported forage resources. Ensiling is a typical and effective method for forage preservation and utilization. While high-quality alfalfa silage faces multiple difficulties due to its low water-soluble carbohydrates, high buffering capacity, and relatively high moisture content at harvest (Ogunade et al. 2018). These make it easier to trigger the undesirable fermentation, resulting in anaerobic deterioration, extensive proteolysis, or an unpleasant rancid odor in alfalfa silage (Zheng et al. 2018). To address these issues, microbial, enzyme, or chemical additives can substantially enhance the likelihood of producing high-quality alfalfa silage.

Cellulase is generally regarded as a multi-enzyme system mainly consisting of endo-β-1,4-glucanase (EC 3.2.1.4), β-glucosidase (EC 3.2.1.21), and exo-β-1,4-glucanase (cellobiohydrolase, EC 3.2.1.74 and EC 3.2.1.91) (Zhang et al. 2024). Currently, cellulase is widely used in ensiled forage with low water-soluble carbohydrate content, which is attributed to its ability to degrade cellulose and hemicellulose in the cell wall of forage into sugars that can be used by lactic acid bacteria, thereby producing a large amount of lactic acid and improving fiber structure and digestibility (Khota et al. 2016; Li et al. 2018a; Zhao et al. 2021a). Cellulase can improve the fermentation quality and degrade plant cell wall (Zhang et al. 2023), decrease pH and ammonia nitrogen production, raise lactic acid concentration in mixed silage of alfalfa and *Leymus chinensis* (Si et al. 2023), increase the abundance of *Lactiplantibacillus* in mixed silage of amaranth and rice straw (Mu et al. 2020). Furthermore, little information is available on the effects of cellulase on alfalfa silage quality, microbial succession, and predicted functional profiles in South Korea. Hence, it’s necessary to investigate the regulatory role of cellulase in Korean alfalfa silage production.

In recent years, the pursuit of healthy, environmentally friendly, and sustainable livestock production has become a global trend and an essential direction for the future development of animal husbandry, with feed safety playing a critical role in this transition. Despite the widespread use of some chemical additives to enhance silage fermentation and minimize losses (Gandra et al. 2024; Muck et al. 2018; Yuan et al. 2017), their application is likely to decline under the global shift toward animal health, human safety, and environmental sustainability, highlighting the need for safer and more effective alternatives. Sodium diacetate (SDA), an organic acid-based salt, is generally recognized as safe and has been used as a preservative additive with antimicrobial activity (Commission 2008). SDA readily dissociates into acetic acid, which rapidly acidifies silage biomass, thereby suppressing the growth and activity of undesirable bacteria during the early stages of fermentation (Auerbach & Theobald 2020). Previous studies have demonstrated that SDA has significant influence on minimizing nutrient loss, reducing proteolysis, and improving aerobic stability (Li et al. 2023, 2022; Yuan et al. 2016). Despite these promising results, its application in South Korea remains limited, and its efficacy in alfalfa silage has yet been evaluated locally.

Microbial inoculants have gained popularity as silage additives, giving credit to their recognized safety and effectiveness in improving fermentation quality. The silage quality is distinctly enhanced, typically with lower pH and NH₃-N levels, along with increased lactic acid concentrations by treating with microbial inoculants (Huo et al. 2022; Li et al. 2020; Si et al. 2023). However, the effectiveness of LAB inoculation may depend strongly on species composition because different LAB species can play distinct roles during ensiling. Microbial inoculants alter silage quality through their strain-specific characteristics (Zielińska et al. 2015). Homofermentative LAB, such as *Lactiplantibacillus plantarum*, *Pediococcus pentosaceus*, *Enterococcus faecium*, *Lactobacillus acidophilus*, and *Lactobacillus bulgaricus*, are mainly responsible for high lactic acid production and rapid pH reduction, thereby reducing nutrient losses (Muck et al. 2018; Okoye et al. 2023). *Pedicoccus* and *Enterococcus* mainly initiate early lactic acid fermentation (Bai et al. 2021; Yang et al. 2019). In contrast, heterofermentative LAB, *Lentilactobacillus buchneri*, that produce a high quantity of acetic acid, inhibit yeasts and molds and prevent aerobic deterioration (Okoye et al. 2023). Therefore, comparing single-species and multi-species LAB formulations with different functional characteristics is vital for understanding how inoculant composition influences alfalfa silage fermentation, microbial succession, and predicted functional profiles. Bai et al. (2021) demonstrated that different inoculants and their combinations distinctly alter fermentation quality, microbial succession, and their metabolic pathways during ensiling. Whereas, only a few studies have evaluated the effects of single LAB species or different moisture levels on alfalfa silage, and most of them have focused mainly on fermentation quality and nutritional value(Jung et al. 2024; Soundharrajan et al. 2025). Particularly, it is unclear whether multi-species inoculants provide additional benefits compared with a single *L. plantarum*-based inoculant, and whether the inclusion of early-stage LAB such as *P. pentosaceus* and *E. faecium* or heterofermentative LAB such as *L. buchneri* can further influence bacterial succession and predicted functional profiles.

Therefore, this study compared five commercial LAB formulations representing different inoculation strategies, including a single *L. plantarum* inoculant, multi-species homofermentative LAB combinations, and a formulation containing heterofermentative *L. buchneri*, together with cellulase and sodium diacetate, to systematically evaluate their effects on fermentation quality, chemical composition, bacterial community succession, and predicted functional profiles of alfalfa silage. This study was designed to clarify whether different additive strategies could differentially affect alfalfa silage fermentation and microbial community, thereby providing practical information for improving alfalfa silage production in South Korea.

## Materials and methods

### Forage cultivation and silage making

Alfalfa was cultivated in the experimental field of the PyeongChang Campus of Seoul National University in September 2022. The first-cut alfalfa was harvested at the full-bloom stage in June 2023 and wilted in the field for 24 h. The wilted alfalfa was chopped into 2–3 cm by a forage cutter (SC-7000, Agricultural Machinery, Inc., South Korea). The chopped alfalfa was mixed evenly and randomly divided into eight equal portions with different additives. According to the new classification system proposed by Zheng et al. (2020), *Lactobacillus plantarum* and *Lactobacillus buchneri* are referred to as *Lactiplantibacillus plantarum* and *Lentilactobacillus buchneri*, respectively*,* whereas *Lactobacillus acidophilus* and *Lactobacillus. bulgaricus* remain within the genus *Lactobacillus.* The control group was treated with distilled water (Control). Five different microbial inoculants, namely LP (Cheongmi Bio Co., Ltd. Anseong, Korea, *Lactiplantibacillus plantarum,* 1.5 × 10^1^⁰ CFU/g), TS (Jungnong Bio Co., Ltd. South Korea, *Lactiplantibacillus plantarum* + *Pediococcus pentosaceus,* 1 × 10^1^⁰ CFU/g), SK (Agri-King, Inc., USA, *Lactiplantibacillus plantarum* + *Pediococcus pentosaceus* + *Enterococcus faecium,* 2.5 × 10^1^⁰ CFU/g), KL (Kemin Industries, Inc., USA, *Lactiplantibacillus plantarum* + *Lactobacillus acidophilus* + *Lactobacillus bulgaricus,* 5.5 × 10^1^⁰ CFU/g), or BSM (Provita Supplements GmbH, Germany, *Lactiplantibacillus plantarum* + *Pediococcus pentosaceus* + *Lentilactobacillus buchneri,* 2.5 × 10^11^ CFU/g) were sprayed evenly on the alfalfa at 1 × 10^6^ colony-forming unit (CFU) / g wilted alfalfa. *Trichoderma* cellulase (C1794-10KU, 6.3 units/mg solid, Sigma-Aldrich, St. Louis, MO, USA) was inoculated on the alfalfa at a dosage of 100 mg/kg (CELL). The sodium diacetate (99%; Shanghai Rhawn Chemical Technology, China) was applied at the rate of 6 g/kg wilted weight (SDA). Approximately 400 g of treated alfalfa was placed into plastic vacuum bags and sealed using a vacuum packer (GOV-4040, Gaonpack Co., Korea). A total of 168 silage bags were prepared for eight treatments, with 21 bags assigned to each treatment. The bags were randomly allocated and stored at room temperature (approximately 27 °C) throughout the ensiling period. A destructive sampling design was employed, in which three bags per treatment were randomly selected and opened at 1, 3, 5, 7, 14, 30, and 60 days after ensiling for fermentation characteristics and chemical composition analyses. Additionally, fresh alfalfa (day 0) and silage samples after 60 days of ensiling were subjected to bacterial community analysis using 16S rRNA gene sequencing.

### Chemical component and fermentation profiles analyses

The dry matter (DM) content was analyzed following the method described in Li et al. (2022) and expressed on fresh matter. Crude protein (CP) content was performed using an elemental analyzer (Euro Vector EA3000, EVISA, Milan, Italy) based on the slightly modified Dumas combustion procedure suggested by Simonne et al. (1997). Neutral detergent fiber (NDF) and acid detergent fiber (ADF) were determined sequentially following the method of Van Soest et al. (1991) by an Ankom2000 fiber analyzer (Ankom Technology Corp., Fairport, NY, USA). Sodium sulfite and α-amylase were involved in the NDF analysis. After ADF procedure, acid detergent lignin (ADL) was evaluated by digesting the residue with 72% sulfuric acid for 3 h in a beaker (Technology 2022). The anthrone-sulfuric acid method was used to analyze water-soluble carbohydrates (WSC) levels (Yemm & Willis 1954). Dry matter recovery (DMR) and gas loss (GL) were calculated as reported by Gheller et al. (2021). The 10 g sample was mixed with 90 g of distilled water, shaken for 1 h at room temperature, and kept at 4 °C overnight. The extract was filtered through four layers of cheesecloth, followed by Whatman No. 1 filter paper. The pH value was measured immediately using a calibrated digital pH meter (AB150, Fisher Scientific International, NH, USA). Ammonia nitrogen (NH_3_-N) was determined by phenol-hypochlorite method using UVIDEC-610 spectrophotometer (Jasco, Tokyo, Japan) and expressed as a percentage of total nitrogen (Broderick & Kang 1980). Organic acid concentration (lactic acid, acetic acid, propionic acid, and butyric acid) were quantified by high-performance liquid chromatography (HPLC) system (Agilent Technologies 1260 Infinity, Santa Clara, USA) equipped with a refractive index detector and an Agilent Hi-Plex H column. The mobile phase was 0.005 N H₂SO_4_ with a flow rate of 0.7 mL/min at 60 °C. What’s more, CP, NDF, ADF, ADL, WSC, and organic acid concentration were expressed on dry matter.

### Microbial community analysis

#### DNA extraction and PCR amplification

Total genomic DNA samples were extracted as described by Ali et al. (2020) using the FastDNA Kit for Soil (MP Biomedicals, CA, USA), following the manufacturer’s instructions. The quantity and quality of extracted DNAs were measured using a NanoDrop NC2000 spectrophotometer (Thermo Fisher Scientific, Waltham, MA, USA) and agarose gel electrophoresis, respectively. PCR amplification of the bacterial 16S rRNA genes V3–V4 region was performed using the forward primer 338F (5'-ACTCCTACGGGAGGCAGCA-3') and the reverse primer 806R (5'-GGACTACHVGGGTWTCTAAT-3'). Thermal cycling consisted of initial denaturation at 98 °C for 5 min, followed by 25 cycles consisting of denaturation at 98 °C for 30 s, annealing at 53 °C for 30 s, and extension at 72 °C for 45 s, with a final extension of 5 min at 72 °C.

#### Sequencing and data analysis

The genome sequencing was performed using the Illumina NovaSeq platform. Raw sequence data were demultiplexed using the demux plugin, followed by primer cutting with the cutadapt plugin. Subsequently, the DADA2 plugin was employed for quality filtering, denoising, and chimera removal. The resulting sequences were clustered at 100% sequence similarity to generate amplicon sequence variants (ASVs) and an abundance table. Genus-level taxonomic assignments obtained from the amplicon sequencing data were interpreted in the microbial community analysis, with *Lactiplantibacillus*, *Lentilactobacillus*, and *Lactobacillus* treated as distinct genera. All downstream data analyses were performed using QIIME2 (version 2024.5) (https://docs.qiime2.org/) and R software (version 4.3.3) (https://www.rproject.org/). The metabolic functions of the microbial community were predicted by PICRUSt2 upon MetaCyc (https://metacyc.org/) and KEGG (https://www.kegg.jp/) databases.

### Statistical analysis

Chemical and fermentation data were analyzed with the general linear model procedure in SPSS (IBM SPSS Statistics for Windows, Version 29.0; IBM Corp., Armonk, NY, USA). The effects of additive treatment, ensiling day, and their interaction were analyzed using a two-way ANOVA model according to Eq. 1:

1 $$ {\mathrm{Y}}_{{{\mathrm{ijk}}}} = {\upmu } + {\mathrm{T}}_{{\mathrm{i}}} + {\mathrm{D}}_{{\mathrm{j}}} + \left( {{\mathrm{T}} \times {\mathrm{D}}} \right)_{{{\mathrm{ij}}}} + {\upvarepsilon}_{{{\mathrm{ijk}}}} $$

where Y_ijk_ = observation, μ = mean, T_i_ = effect of treatments (additives), D_j_ = day of ensiling, (T × D)_ij_ = interaction effect of treatment × day of ensiling, and ɛ_ijk_ = error term. Each silage bag was considered an independent experimental unit in the model. The normality of residuals and homogeneity of variances were evaluated using the Shapiro–Wilk test and Levene’s test, respectively. Duncan’s multiple range test was used for mean separation when significant main effects or interactions were detected by ANOVA. Duncan’s test was selected to compare multiple additive treatments within the same experimental design. Differences were considered significant at *p* < 0.05. Microbial community analyses were performed using QIIME2 (version 2024.5) and R software (version 4.3.3) based on the ASV table. The ASV table was rarefied to the same sequencing depth before diversity analysis. Differences in alpha diversity indices among treatments were analyzed using the Kruskal–Wallis test. Beta diversity was assessed using PCoA based on Bray–Curtis dissimilarity, and differences in bacterial community composition among treatments were evaluated by permutational multivariate analysis of variance (PERMANOVA). The relative abundance of dominant bacterial taxa was compared among treatments using the Kruskal–Wallis test. For linear discriminant analysis effect size (LEfSe), the Kruskal–Wallis test was used to detect taxa with significant differences among groups, followed by linear discriminant analysis (LDA) to estimate the effect size of each differentially abundant taxon. Taxa with an LDA score > 3.0 and *p* < 0.05 were considered significantly enriched.

## Results and discussion

### The chemical composition of alfalfa prior to ensiling

Before ensiling, the DM content in alfalfa was 35.01%, which is within the recommended range 30–40% (Yuan et al. 2017). The NDF, ADF, and CP were 50.42%, 33.37%, and 18.46% DM, respectively. The WSC content in this experiment (5.98% DM) was only slightly above the minimum threshold of 5% DM required for normal fermentation (Ni et al. 2018), indicating a potential risk of undesirable fermentation.

### The chemical characteristics of alfalfa silage during ensiling

Additives, ensiling days, and their interactions had a significant effect on DM, DMR, GL, and CP levels as described in Table 1 shown. The DM content showed a downward trend with the prolongation of ensiling days in all groups, which may be attributed to the loss of organic matter and gas production during ensiling (*p* < 0.05). There was significantly higher DMR in KL, LP, or SDA-treated alfalfa compared to the control group (*p* < 0.05), while no difference was found in BSM-inoculated alfalfa. Although studies displayed that LAB inoculation has the ability to significantly improve DMR of alfalfa and other forages, the specifics depend on the type of LAB inoculation (Oliveira et al. 2017). Mu et al. (2020) announced that *L. plantarum* group improves the DMR of mixed amaranth and rice straw silage, which agrees with our result.

**Table 1:** Chemical compositions, gas loss, and dry matter recovery in alfalfa silage treated with different additives during ensiling

Items	Additive	Ensiling days							SEM	*p*-value		
		D1	D3	D5	D7	D14	D30	D60		D	A	D × A
DM (%)	C	31.58^bB^	32.10^aA^	31.37^bBC^	31.29^bBC^	31.55^bB^	30.79^cCD^	30.17^dE^	0.04	< 0.001	< 0.001	0.024
	SK	31.57^aB^	31.51^aB^	31.05^bBC^	31.35^abBC^	31.31^abBC^	31.30^abBC^	31.44^abB^				
	TS	31.15^abB^	31.61^aB^	31.10^abBC^	31.18^abCD^	31.08^abCD^	31.26^abBC^	30.73^bCDE^				
	BSM	31.58^aB^	31.29^abB^	31.16^abBC^	31.27^abBC^	30.85^abD^	30.46^bD^	30.58^bDE^				
	LP	31.32^aB^	31.42^aB^	30.90^bC^	30.95^bD^	30.91^bD^	31.06^abBCD^	31.22^abBC^				
	KL	31.49^B^	31.64^B^	31.56^B^	31.58^B^	31.58^B^	31.54^AB^	31.29^BC^				
	CELL	31.38^aB^	31.39^aB^	31.20^ab^	31.15^abCD^	31.29^abBC^	31.15^abBCD^	30.84^bBCD^				
	SDA	32.36^abA^	32.33^abA^	32.19^abA^	32.54^aA^	32.12^abA^	32.03^bA^	32.11^bA^				
DM recovery	C	89.34^bABC^	90.75^aA^	88.11^cBC^	87.89^cC^	88.40^cA^	85.56^dC^	85.43^dDE^	0.12	< 0.001	< 0.001	< 0.001
	SK	88.38^aC^	87.93^abC^	86.56^cC^	87.34^abcC^	86.98^bcB^	86.69^cB^	87.10^bcBC^				
(%)	TS	90.11^abA^	90.58^aAB^	89.09^abcAB^	89.31^abB^	88.79^bcA^	89.01^abcA^	87.48^cBC^				
	BSM	88.22^aC^	87.18^aC^	86.81^abC^	87.01^aC^	85.46^bcC^	85.06^cC^	84.38^cE^				
	LP	88.90^abBC^	89.37^aB^	87.87^bcBC^	87.79^bcC^	87.42^cB^	87.53^cB^	88.10^bcAB^				
	KL	89.75^AB^	89.89^AB^	89.59^A^	89.59^B^	89.35^A^	88.94^A^	88.37^AB^				
	CELL	88.17^aC^	88.05^abC^	87.40^abcC^	87.00^bcdC^	87.38^abcB^	86.65^cdB^	86.22^dCD^				
	SDA	90.54^aA^	90.31^aAB^	89.77^abA^	90.72^aA^	88.98^bcA^	88.71^cA^	89.16^bcA^				
Gas loss	C	0.29^cBC^	0.31^cB^	0.50^bA^	0.50^bA^	0.57^bA^	0.80^aA^	0.77^aA^	0.01	< 0.001	< 0.001	< 0.001
	SK	0.22^dC^	0.31^cB^	0.34^cB^	0.35^cD^	0.44^bC^	0.52^aC^	0.51^aD^				
	TS	0.33^cAB^	0.38^cA^	0.39^bcAB^	0.40^bcCD^	0.47^bBC^	0.56^aC^	0.57^aC^				
	BSM	0.29^ dB^	0.36^cAB^	0.37^cB^	0.40^cC^	0.52^bAB^	0.63^aB^	0.63^aB^				
	LP	0.37^bcA^	0.30^cB^	0.32^cB^	0.39^bcCD^	0.47^abBC^	0.57^aC^	0.54^aCD^				
	KL	0.05^dD^	0.15^cC^	0.17^cC^	0.19^cE^	0.26^bD^	0.35^aD^	0.31^abE^				
	CELL	0.28^dBC^	0.33^cAB^	0.36^cB^	0.45^bB^	0.45^bC^	0.56^aC^	0.56^aC^				
	SDA	0.04^cD^	0.09^cD^	0.13^cC^	0.14^cF^	0.32^aD^	0.33^aD^	0.25^bF^				
CP	C	17.30^aAB^	17.12^abA^	16.43^abAB^	16.17^abcB^	15.81^bcB^	15.36^cdC^	14.53^dC^	0.21	< 0.001	< 0.001	< 0.001
(% DM)	SK	16.61^AB^	16.46^B^	16.27^AB^	16.90^A^	16.21^AB^	16.33^AB^	16.81^B^				
	TS	17.71^A^	17.23^A^	16.62^AB^	16.97^A^	16.59^AB^	16.72^AB^	16.61^B^				
	BSM	17.13^AB^	16.43^B^	16.05^AB^	16.54^AB^	16.52^AB^	16.45^AB^	16.40^B^				
	LP	16.57^abAB^	16.40^abB^	16.48^abAB^	16.35^abB^	16.14^bAB^	17.17^aA^	17.09^abAB^				
	KL	17.49^AB^	17.25^A^	17.01^A^	16.92^A^	16.69^A^	16.60^AB^	16.81^B^				
	CELL	16.52^aB^	15.19^bC^	15.72^abB^	16.06^abB^	16.17^abAB^	15.87^abBC^	16.14^abB^				
	SDA	17.14^abAB^	16.85^bcAB^	16.09^cAB^	16.98^bcA^	16.74^bcA^	16.83^bcAB^	17.96^aA^				

DM, dry matter; CP, crude protein; ^a−d^ Means within rows with unlike superscripts differ (*p* < 0.05); ^A−E^ Means within columns with unlike superscripts differ (*p* < 0.05); C, control: no additives; LP, *Lactiplantibacillus plantarum*; TS, *Lactiplantibacillus plantarum* + *Pediococcus pentosaceus*; SK, *Lactiplantibacillus plantarum* + *Pediococcus pentosaceus* + *Enterococcus faecium*; KL, *Lactiplantibacillus plantarum* + *Lactobacillus acidophilus* + *Lactobacillus bulgaricus*; BSM, *Lactiplantibacillus* + *Pediococcus pentosaceus* + *Lentilactobacillus buchneri*; CELL, *Trichoderma* cellulase, SDA, sodium diacetate. SEM, standard error of the mean. D, ensiling days, A, additive treatment, D × A, the interaction of ensiling days and additive treatment

Additionally, it was in accordance with the lower GL in KL and SDA, and SDA consistently had the highest DM content throughout the ensiling process (*p* < 0.05). This can be explained by the fact that SDA is readily ionized to acetic acid, which immediately acidifies the forage biomass, thereby inhibiting the growth and activity of undesirable bacteria in the early stages of fermentation and minimizing nutrient losses and gas production during ensiling (Wen et al. 2017). The most severe DM or GL is often caused by *Clostridium*, which converts lactic acid or sugars into butyric acid and CO₂, H₂, and NH₃, resulting in a 51% DM loss (Muck 2010). However, butyric was not present in our study, so it can be concluded that there was limited or undetectable *clostridial* fermentation. Aerobic bacteria metabolize soluble carbohydrates and lactic acid to produce CO₂, but a rapid drop in pH inhibits their growth and activity during the early ensiling phase (Pahlow et al. 2003). Additionally, heterofermentation utilizes lactic acid or sugars to produce acetic acid and CO₂ (McDonald et al. 1991). Therefore, it can be inferred that GL may be mainly caused by CO₂ production. Kung et al. (2003) manifested that the CO₂ production in silage is affected by additives. Our experimental results found that all treatment groups could significantly reduce GL (*p* < 0.05), which may be mainly due to the inhibition of CO₂ production. BSM had higher GL and lower DMR than TS and SK (*p* < 0.05), suggesting that formulations containing *L. buchneri* may be more prone to gas loss and dry matter losses. It’s hypothesized that this is due to heterofermentation during the later stages of ensiling. KL treatment exhibited markedly lower GL than the LP, SK, and TS groups during ensiling, suggesting that the combination of *L. plantarum*, *L. acidophilus*, and *L. bulgaricus* was more effective in minimizing gas production, possibly due to their synergistic effect. This may be attributed to the consistent metabolic pathways among the three LAB strains, which are more conducive to maintaining an efficient unidirectional carbon flow towards lactic acid, resulting in rapid acidification and reducing the oxidation of sugars or lactic acid into CO₂, H₂, or other volatile products. The CP level was not distinctly altered in SK, TS, and KL groups during ensiling, indicating that fermentation is successful in preserving the protein, attributed to a sharp decline in pH. Nevertheless, a greater downward trend was found in the CELL and BSM groups compared to other treatments due to their slower pH drop. By day 60, all additives appeared to have significantly higher CP content compared to control group (*p* < 0.05), suggesting that additives optimize protein preservation.

Although studies have reported lower aNDF and ADF in alfalfa silage treated with *L. plantarum* + *P. pentosaceus* or *L. plantarum* + *P. pentosaceus* + *E. faecium* (Bai et al. 2021), no similar results were found in this experiment. This difference may be related to epiphytic flora, environment, or strain origin. CELL-treated silage displayed markedly lower NDF and ADF (*p* < 0.05), whereas ADL was not significantly altered across groups (Table 2), indicating that cellulase can effectively degrade structural carbohydrates. This result concurs with the findings of Si et al. (2023) and Xiong et al. (2022), who similarly noted notable reductions in NDF and ADF following cellulase application.

**Table 2:** Fiber composition in alfalfa silage treated with different additives during ensiling

Items	Additive	Ensiling days							SEM	*p*-value		
		D1	D3	D5	D7	D14	D30	D60		D	A	D × A
NDF	C	49.36^aA^	47.42^bA^	47.58^abA^	47.82^abA^	47.22^bA^	47.00^bA^	47.10^bA^	0.62	< 0.001	< 0.001	0.15
(% DM)	SK	48.34^aAB^	47.08^abA^	47.18^abA^	46.78^abA^	47.55^aA^	44.58^bB^	46.84^abA^				
	TS	48.28^AB^	47.13^A^	46.94^A^	46.93^A^	47.50^A^	46.69^A^	47.97^A^				
	KL	47.26^BC^	47.62^A^	45.86^A^	46.17^A^	46.28^A^	47.27^A^	47.14^A^				
	BSM	48.77^AB^	47.21^A^	47.27^A^	46.93^A^	47.17^A^	47.42^A^	46.97^A^				
	LP	48.71^aAB^	46.97^abA^	46.77^abA^	45.78^bA^	46.07^bA^	45.74^bAB^	46.83^abA^				
	CELL	46.32^aC^	44.74^bB^	43.09^cB^	42.62^cdB^	41.80^cdB^	41.72^cdC^	41.36^ dB^				
	SDA	48.74^aAB^	46.57^abA^	47.50^abA^	46.45^abA^	45.84^bA^	47.25^abA^	47.02^abA^				
ADF	C	31.95	31.27^A^	30.79^A^	31.87^A^	32.93^A^	31.97^A^	32.38^A^	0.60	< 0.001	0.31	0.56
(% DM)	SK	30.88	30.86^A^	30.70^A^	30.75^A^	31.69^AB^	32.05^A^	30.51^C^				
	TS	31.35	30.57^A^	31.11^A^	31.75^A^	31.05^AB^	30.72^A^	31.97^A^B				
	KL	31.18	30.29^A^	30.52^A^	31.02^A^	30.09^B^	31.39^A^	30.73^BC^				
	BSM	31.69^abc^	30.50^cA^	31.74^abcA^	32.48^aA^	31.10^bcAB^	31.63^abcA^	32.01^abAB^				
	LP	30.33	31.13^A^	30.98	31.50^A^	30.56^B^	30.51^A^	30.54^C^				
	CELL	29.96^a^	28.25^abB^	28.59^abB^	27.92^bB^	27.67^bC^	27.23^bB^	27.61^bD^				
	SDA	30.82	30.25^A^	31.07^A^	31.34^A^	30.63^B^	31.69^A^	31.11^ABC^				
ADL	C	8.72	8.49^AB^	8.80	9.02	9.13	9.10^AB^	8.66	0.43	0.15	0.002	0.72
(% DM)	SK	8.63	8.40^AB^	9.49	8.80	9.03	8.51^AB^	8.75				
	TS	8.45	8.45^AB^	9.10	8.05	9.44	8.99^AB^	9.15				
	KL	8.19	7.91^B^	8.24	7.78	8.84	9.24^AB^	9.28				
	BSM	8.86	8.43^AB^	8.99	8.60	9.45	9.80^A^	8.70				
	LP	9.05	9.12^A^	8.65	8.97	9.11	8.58^AB^	8.64				
	CELL	8.13	8.11^AB^	8.34	8.88	8.69	8.01^B^	8.97				
	SDA	8.67	8.10^AB^	9.61	8.77	9.34	9.53^AB^	8.99				

DM, dry matter; NDF, neutral detergent fiber; ADF, acid detergent fiber; ADL, acid detergent lignin; ^a−d^ Means within rows with unlike superscripts differ (*p* < 0.05); ^A−D^ Means within columns with unlike superscripts differ (*p* < 0.05); C, control: no additives; LP, *Lactiplantibacillus plantarum*; TS, *Lactiplantibacillus plantarum* + *Pediococcus pentosaceus*; SK, *Lactiplantibacillus plantarum* + *Pediococcus pentosaceus* + *Enterococcus faecium*; KL, *Lactiplantibacillus plantarum* + *Lactobacillus acidophilus* + *Lactobacillus bulgaricus*; BSM, *Lactiplantibacillus* + *Pediococcus pentosaceus* + *Lentilactobacillus buchneri*; CELL, *Trichoderma* cellulase, SDA, sodium diacetate. SEM, standard error of the mean. D, ensiling days, A, additive treatment, D × A, the interaction of ensiling days and additive treatment

### Discrepancy in fermentation profiles of alfalfa silage

**Fig. 1:**
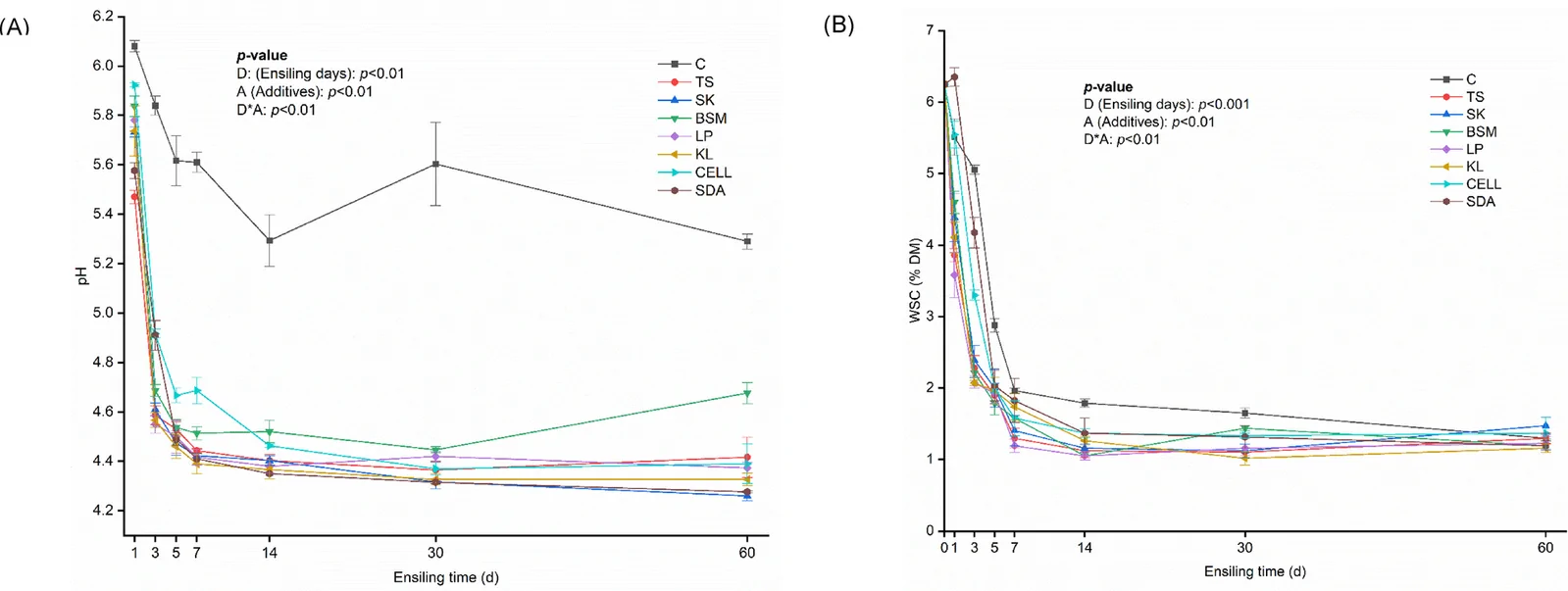
Kinetics of pH value and WSC concentration in alfalfa silage treated with different additives during ensiling. (A) Kinetics of pH value during ensiling. (B) Kinetics of Water-soluble carbohydrate (WSC) during ensiling. C, control: no additives; LP, *Lactiplantibacillus plantarum*; TS, *Lactiplantibacillus plantarum* + *Pediococcus pentosaceus*; SK, *Lactiplantibacillus plantarum* + *Pediococcus pentosaceus* + *Enterococcus faecium*; KL, *Lactiplantibacillus plantarum* + *Lactobacillus acidophilus* + *Lactobacillus bulgaricus*; BSM, *Lactiplantibacillus* + *Pediococcus pentosaceus* + *Lentilactobacillus buchneri*; CELL, *Trichoderma* cellulase, SDA, sodium diacetate. Error bars indicate, standard error of the mean

**Fig. 2:**
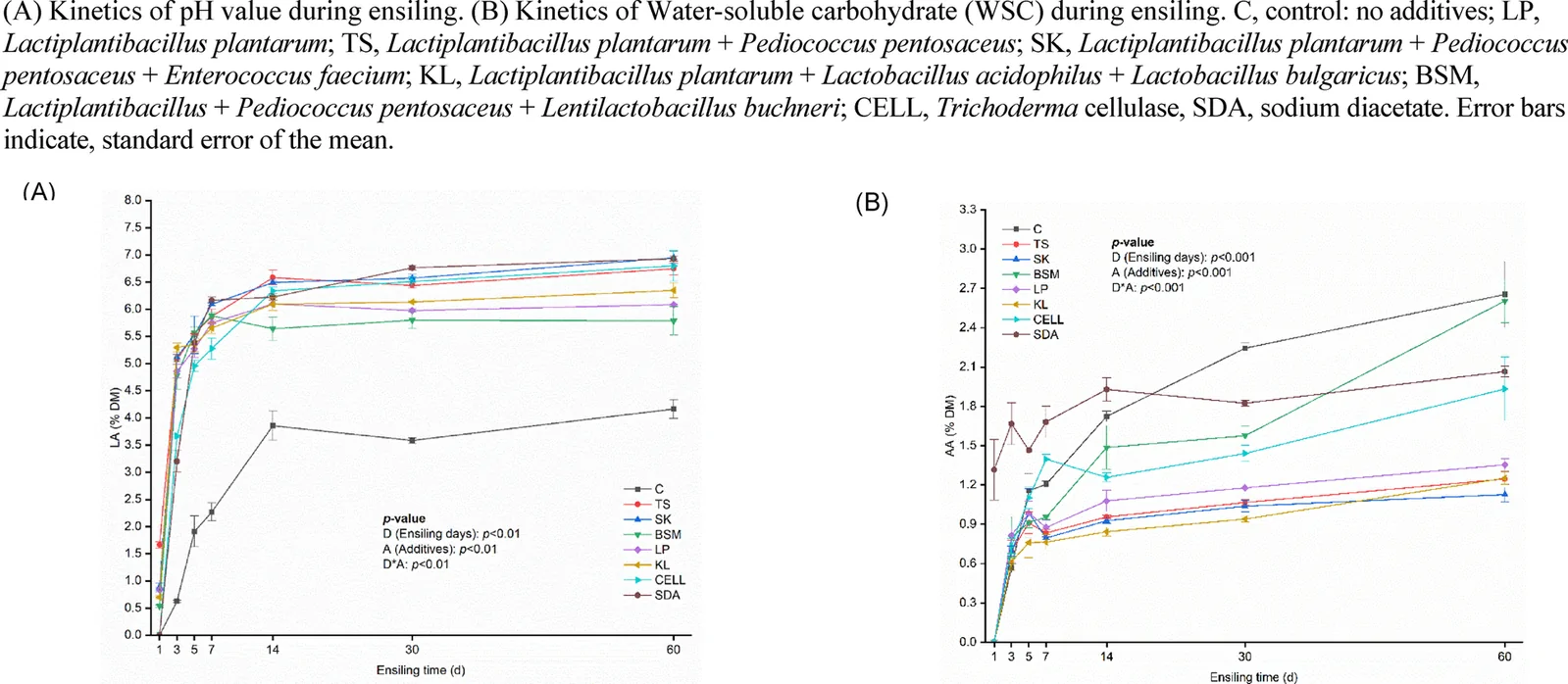

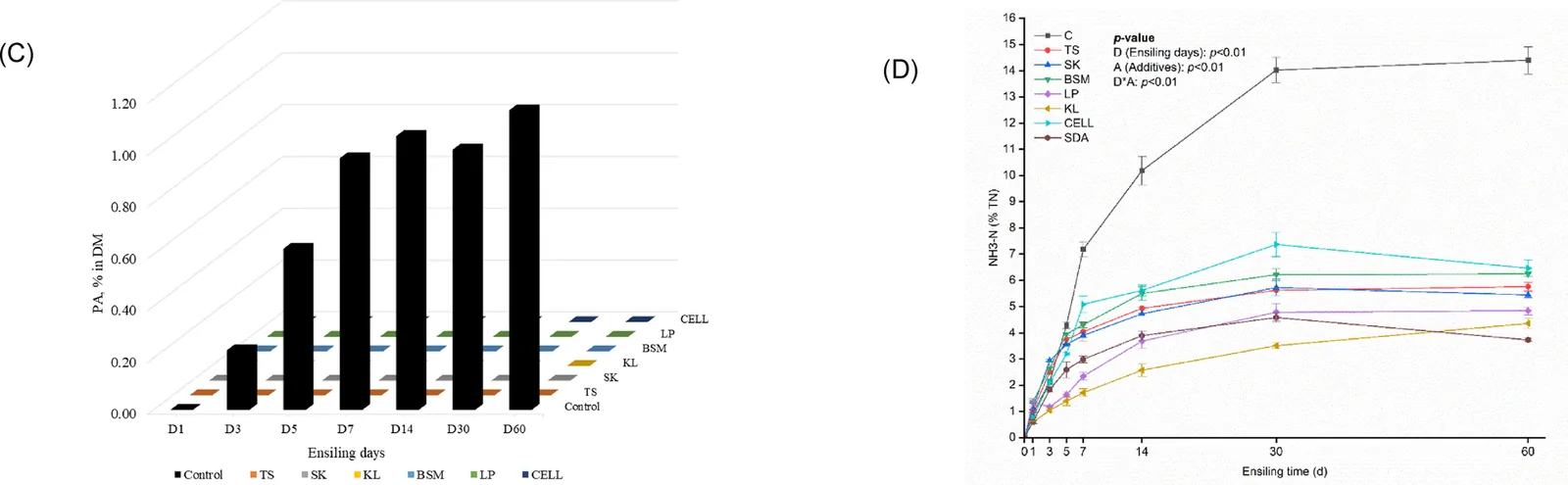
Changes in organic acid concentrations and NH_3_–N level in alfalfa silage treated with different additives during ensiling. (A) Lactic acid (LA) concentration. (B) Acetic acid (AA) concentration. (C) Propionic acid (PA) concentration. (D) Ammonia–nitrogen (NH₃–N) concentration. C, control: no additives; LP, *Lactiplantibacillus plantarum*; TS, *Lactiplantibacillus plantarum* + *Pediococcus pentosaceus*; SK, *Lactiplantibacillus plantarum* + *Pediococcus pentosaceus* + *Enterococcus faecium*; KL, *Lactiplantibacillus plantarum* + *Lactobacillus acidophilus* + *Lactobacillus bulgaricus*; BSM, *Lactiplantibacillus* + *Pediococcus pentosaceus* + *Lentilactobacillus buchneri*; CELL, *Trichoderma* cellulase, SDA, sodium diacetate. Error bars indicate, standard error of the mean

In our experiment, the pH levels decreased throughout the ensiling process, with a significantly faster decline observed during the first 5 days except for the control group, and pH levels slowly tended to stabilize after 14 days of ensiling (Fig. 1A), which coincides with the studies of Desta et al. (2016) and Si et al. (2023). Rapid initial acidification is effective in controlling the growth of *enterobacteria* and *Clostridia* to ensure successful fermentation (Pahlow et al. 2003). Thus, the treatment groups likely suppress the growth and metabolism of undesirable bacteria by acidifying the environment in the early stage. Except for the control group (5.29) and the BSM group (4.68), the pH values of all other groups were lower than 4.5 after 60 days of ensiling, which is regarded as good-quality alfalfa silage (Kung et al., 2018). This is consistent with previous studies that LAB inoculants are effective to improve the silage quality of various forages (Jung et al. 2024; Li et al. 2023; Mu et al. 2020). The decrease in pH is mainly the result of LAB fermenting WSC to produce lactic acid in an anaerobic environment (McDonald et al. 1991). Furthermore, WSC may also have the possibility to be consumed by other metabolic pathways, such as by undesirable *Clostridium*, molds, *enterobacteria*, or yeasts, or by plant respiration in the early stages of ensilage (Pahlow et al. 2003). In this study, the decreasing trend of WSC concentration was consistent with the pH value, which decreased as the fermentation time lengthened and was primarily concentrated in the first 5 days (approximately 66.56% of WSC consumption) in additive groups (Fig. 1B). Thus, it indicated that the process was dominated by LAB to mainly convert WSC into lactic acid. This is further confirmed by the fact that lactic acid concentration in treatment group was higher and accumulated rapidly in the first 5 days (Fig. 2A), and no propionic acid and butyric acid were detected. This also indicated that there was limited *clostridial* metabolism in silage treated with additives.

The lactic acid content was recorded as the highest among all organic acids, which is also the key justification for the decrease in pH value in this study (Fig. 2A). *Cocci*-type LAB were confirmed to dominate in the early stages of ensiling, rapidly creating an acidifying fermentation environment (Pahlow et al. 2003). This study found that although all silages inoculated with LAB displayed significantly faster lactic acid accumulation and pH decline compared to the control group, TS (*L. plantarum* + *P. pentosaceus*) was most prominent on the first day (1.66% DM). This phenomenon suggested that the combination of *L. plantarum* and *P. pentosaceus* has a more outstanding effect in accelerating lactic acid fermentation, consistent with the findings of Bai et al. (2021). Furthermore, it may indicate that the key to accelerating silage acidification lies not only in the number of inoculated bacteria, but also more in the functional complementarity between strains. Multi-strain inoculum can lead to resource competition, metabolic conflicts, and inter-strain antagonism, which may be found in BSM (*L. plantarum* + *P. pentosaceus* + *L. buchneri*), resulting in the slowest pH decrease (4.68) and lactic acid (5.78% DM) among the five LAB inoculants (*p* < 0.05). This may be attributed to *L. buchneri* is a heterofermentative bacterium, its metabolism conflicts with the other two homofermentative strains, as well as competition for fermentation substrates. Additives significantly increased the lactic acid levels, among which TS (6.75% DM), SK (6.95% DM), KL (6.35% DM), and SDA (6.93% DM) were more outstanding (*p* < 0.05) after 60 days (Fig. 2A). This may be due to the fact that sufficient homofermentative LAB promoted homo-fermentation, while SDA increased the utilization efficiency of WSC by epiphytic LAB because acetic acid effectively inhibited undesirable bacteria in the early stage (Jung et al. 2024; Li et al. 2018a). Previous studies demonstrated that as the application level of SDA increased, the lactic acid content increases, while at 9 g/ kg, it decreases, but the number of LAB is not affected (Yuan et al. 2016). After ensiling, TS had a higher lactic acid concentration and pH value than KL, so we speculated that this might be due to its higher NH_3_–N level neutralizing the produced organic acid. On day 1, neither lactic acid nor acetic acid was detected in the control and CELL groups, which was probably due to the low number or weak activity of epiphytic LAB, which could not effectively launch lactic acid fermentation.

Interestingly, acetic acid (1.32% DM) was only detected in the SDA group, but no lactic acid on the first day (Fig. 2B), coinciding with a descent in pH. Hence, it is speculated that the acidification process is initiated, not relying on the production of lactic acid, but directly from the acetic acid released and dissociated by SDA. Studies have shown that acetic acid has strong antibacterial properties (Auerbach & Theobald 2020). So this process likely establishes a low pH environment at the initial stage of ensiling, which is conducive to the selective expansion of subsequent LAB and may reduce the initial non-target degradation of sugars and proteins. The acetic acid level sharply increased after 7 days of ensiling and yielded the highest after 60 days for the control (2.65% DM) and BSM (2.60% DM) groups (*p* < 0.05) (Fig. 2B). Acetic acid is generally produced by heterofermentative LAB, *propionibacterium*, or *enterococcus*, and may also come from chemical additives (McDonald et al. 1991). Firstly, this may be on account of the failure of high pH to control the metabolism of propionic bacteria or *enterococci* in the control group, and it may also be due to the shift of homo-fermentation to hetero-fermentation. Studies have shown that low-sugar legumes frequently give rise to a transformation in fermentation type (Li et al. 2018b). But for BSM, it is caused by the heterofermentative *L. buchneri,* which typically ferments lactic acid or WSC to acetic acid (Pahlow et al. 2003). This hypothesis was further confirmed by the decreased lactic acid level in the BSM group. Propionic acid was absent in all additive groups, in keeping with the study that in a low pH environment, propionic bacteria cannot grow or their activity is inhibited (Muck 2013). Besides, the propionic acid concentration in the control group exceeded the acceptable range (1% DM, Fig. 2C) (Agarussi et al. 2019) and pH was greater than 5, indicating that untreated alfalfa could not be successfully fermented.

As shown in Fig. 2D, the control group had the largest and fastest increase in NH_3_–N (*p* < 0.05). After 60 days, it reached 14.40% TN (144 g/kg TN) in the control group, outdistancing the limit concentration of 100 g/kg for well-fermented silages (Mu et al. 2020). This could be speculated to be caused by the action of plant proteolytic enzymes and undesirable bacteria (Ogunade et al. 2018). In our experiment, NH_3_-N concentration was markedly lowered in the alfalfa silage treated with additives compared with the control group, with KL (4.35% TN) and SDA (3.72% TN) being the lowest (*p* < 0.05), indicating that the additive treatments significantly inhibit NH_3_-N production. Our experimental result is in accord with previous studies (Li et al. 2023; Mu et al. 2020; Si et al. 2023). *L. plantarum* rapidly consumes WSC to generate large amounts of lactic acid, causing a fast drop in pH and inhibiting the activity of proteolytic bacteria and plant proteases (McDonald et al. 1991). Studies have clearly indicated that *L. acidophilus* and *L. bulgaricus* possess sophisticated proteolytic systems and peptide or amino acid transport and assimilation mechanisms (Altermann et al. 2005). Therefore, they may reduce the terminal conversion of nitrogen to NH₃–N by assimilating released peptides and free amino acids into bacterial proteins. In this study, KL had the lowest NH_3_-N concentration throughout the ensiling process, (*p* < 0.05) which may result from the synergistic effect of rapid acidification by *L. plantarum* and the nitrogen assimilation capabilities of *L. acidophilus* and *L. bulgaricus.* However, peptide uptake, amino acid assimilation, and bacterial protein synthesis were not directly measured in the present study and should be further investigated in future research. The NH_3_-N concentrations in SK (5.44% TN), TS (5.76% TN), and BSM (6.24% TN) groups were higher than in LP (4.84% TN) group, indicating that there may be certain antagonistic effect as adding *P. pentosaceus* and *E. faecium*, or *L. buchneri* to *L. plantarum* inoculation alone. However, an interesting phenomenon occurred among SK, TS, and BSM that the NH_3_-N levels were SK > BSM > TS during the first 3 days of ensiling, but from day 5 onwards, the concentrations shifted to BSM > TS ≈ SK. Studies have shown that *E. faecium* maintains high metabolic activity at pH > 5.0 (Morandi et al. 2005), participating in protein degradation through extracellular proteases and various peptidases (Jeong et al. 2014). While the pH drops below 4.5, its growth and metabolism are significantly inhibited or even stopped (Vaningelgem et al. 2006). In this experiment, pH did not achieve the inhibition threshold in the early stage of silage (6.0–4.6), *E. faecium* in the SK treatment remained active, accelerating protein degradation along with endogenous plant proteases and other bacteria, thus promoting early NH₃–N accumulation. In the BSM treatment, the presence of the heterofermentative *L. buchneri* converted available sugars or lactic acid into acetic acid, thereby slowing the decline in pH. As results, plant proteases and undesirable bacteria remained active for a longer period, ultimately resulting in higher NH₃–N accumulation. By day 5, NH₃–N concentrations in the SK and TS tended to converge, attributed to the pH being below 4.5, where metabolic activity of *E. faecium* was strongly inhibited. The BSM displayed higher NH₃–N level due to heterofermentative characteristics of *L. buchneri*, consistent with its slow pH decrease.

### Bacterial diversity and community analysis during ensiling

**Fig. 3:**
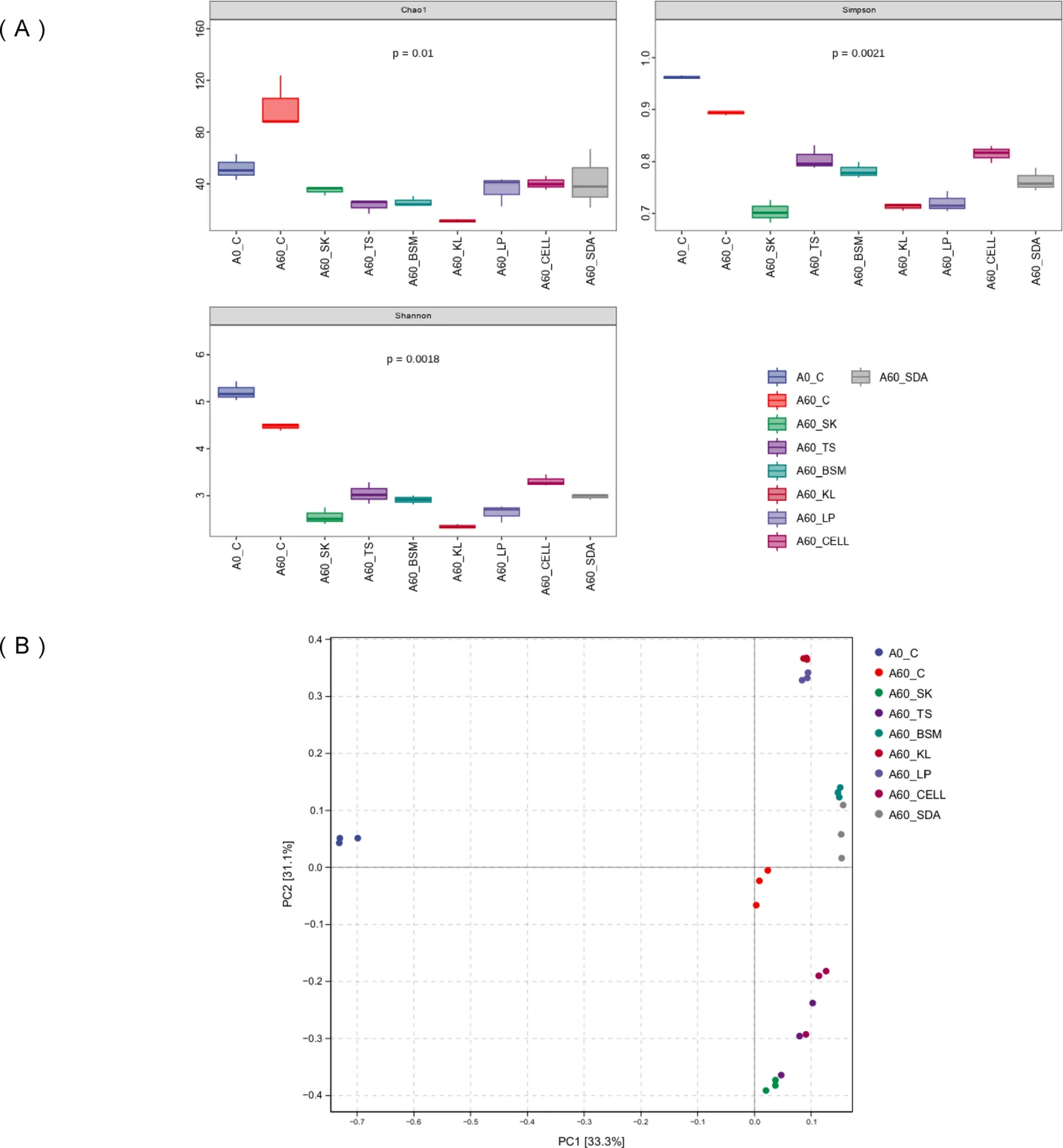
Alpha and beta diversity of bacterial communities in fresh alfalfa and alfalfa silage treated with different additives after 60 days of ensiling. (A) Alpha diversity indices, including Chao1, Simpson, and Shannon indices. (B) Principal coordinate analysis (PCoA) based on Bray–Curtis dissimilarity of bacterial community composition. A0, fresh alfalfa; A60, alfalfa silage after 60 days of ensiling; C, control: no additives; LP, *Lactiplantibacillus plantarum*; TS, *Lactiplantibacillus plantarum* + *Pediococcus pentosaceus*; SK, *Lactiplantibacillus plantarum* + *Pediococcus pentosaceus* + *Enterococcus faecium*; KL, *Lactiplantibacillus plantarum* + *Lactobacillus acidophilus* + *Lactobacillus bulgaricus*; BSM, *Lactiplantibacillus* + *Pediococcus pentosaceus* + *Lentilactobacillus buchneri*; CELL, *Trichoderma* cellulase, SDA, sodium diacetate

In this study, the V3-V4 region of the 16S rRNA gene was adopted to probe into the bacterial diversity in fresh or ensiled alfalfa. After a series of filtering, denoising, and chimera removal steps, a total of 1,491,487 high-quality sequences were acquired from 27 samples, with an average of 55,240 sequences per sample. To ensure comparable sequencing depth among samples, the feature table was rarefied to 15,000 reads per sample for alpha- and beta-diversity analyses. The difference in diversity and richness for fresh or ensiled alfalfa is displayed in Fig. 3A. After ensiling, the Shannon index and Simpson index of all alfalfa silage were dramatically reduced (*p* < 0.05), and the additive-treated group revealed a lower value than the control, with KL having the lowest (*p* < 0.05). This is in line with the study of Mu et al. (2020) and Ren et al. (2020). This demonstrated that both chemical additives and microbial inoculants promoted the transformation of complex and diverse microbial communities into a simplified diversity LAB-dominant structure. Lower bacterial diversity is typically present in high-quality silage (Muraro et al. 2021). Therefore, it can be inferred that additives improved the quality of alfalfa silage, among which KL was the most outstanding. There was a phenomenon that the Chao1 value had a significant decrease in additive groups compared with fresh alfalfa (*p* < 0.05), consistent with the results of Ogunade et al. (2018) and Ren et al. (2020), while a lift was observed in the control group. Additionally, the lowest Chao1 was recorded in KL, only 11.28, which was much lower than other groups (*p* < 0.05), suggesting possible functional complementarity among *L. plantarum*, *L. acidophilus* and *L. bulgaricus*.

Principal coordinates analysis (PCoA) illustrated differences in microbial community segregation patterns among treatments (Fig. 3B). Treatment had a significant effect on bacterial community composition based on Bray–Curtis distances, as confirmed by PERMANOVA analysis (F = 11.7711, R^2^ = 0.8395, *p* = 0.001). PC1 and PC2 explained 33.3% and 31.1% of the community variation, respectively, indicating that the analysis has good interpretability. The pre-ensiling sample and all post-ensiling samples were obviously separated, indicating that a significant microbial community succession occurred after ensiling (*p* < 0.05). Our result is similar to these studies (Liu et al. 2024; Mu et al. 2020). Liu et al. (2024) expressed that LAB treatments altered microbial community structure. LP and KL were significantly clustered in the upper right region, possibly reflecting that *L. plantarum* still dominated in KL, making its community structure almost identical to that of *L. plantarum* alone. TS and SK, containing *P. pentosaceus* or *E. faecium*, clustered in the lower right region, suggesting the rapid proliferation of *P. pentosaceus* in early stage and the addition of *E. faecium*, which may alter the community succession pathway, causing it to deviate from *L. plantarum*-dominated structure. BSM, due to the involvement of *L. buchneri*, formed a unique community structure significantly separated from TS, resembling SDA. *L. buchneri* is a typical heterofermentative LAB, which metabolizes lactic acid to produce acetic acid and 1,2-propanediol may lead to a significant deviation of the community structure from a purely homofermentation pathway in the later stages. Our results are equivalent to those reported by Bai et al. (2021), which represented obvious differences in bacterial community succession with different inoculants. SDA tended to be in the central region, indicating its primary function is to suppress aerobic bacteria, yeast, or undesirable bacteria rather than actively reshaping the microbial community. CELL were positioned between the control and TS. It can be explained by CELL improved fermentation by promoting fiber degradation and increasing the release of fermentable substrates, resulting in a microbial structure that differed from the control group and was biased towards TS.

### Microbial community composition in alfalfa silage treated with different additives

**Fig. 4:**
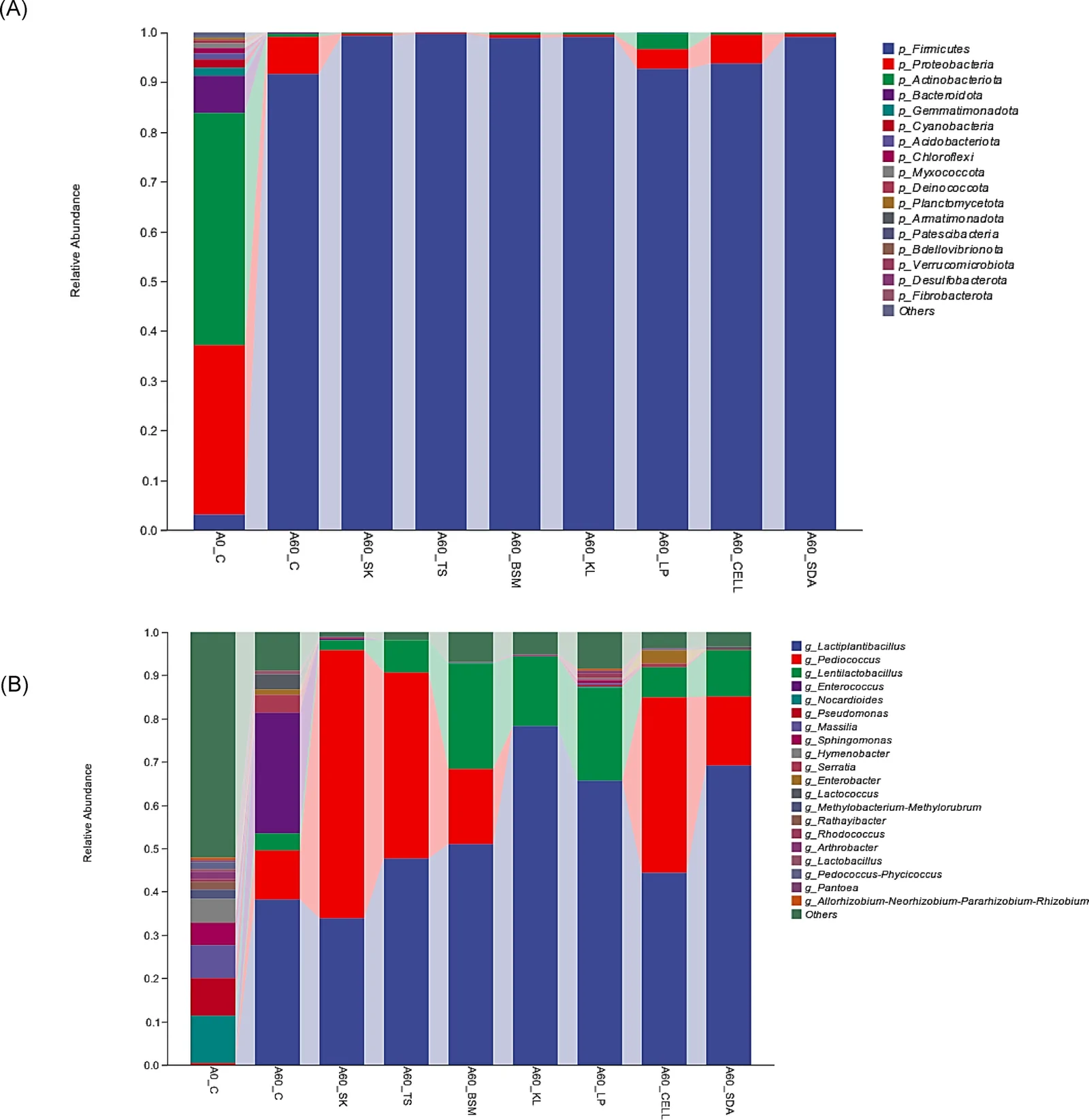
Bacterial community composition of fresh alfalfa and alfalfa silage treated with different additives after 60 days of ensiling. (A) Relative abundance of bacterial communities at the phylum level. (B) Relative abundance of bacterial communities at the genus level. A0, fresh alfalfa; A60, alfalfa silage after 60 days of ensiling; C, control: no additives; LP, *Lactiplantibacillus plantarum*; TS, *Lactiplantibacillus plantarum* + *Pediococcus pentosaceus*; SK, *Lactiplantibacillus plantarum* + *Pediococcus pentosaceus* + *Enterococcus faecium*; KL, *Lactiplantibacillus plantarum* + *Lactobacillus acidophilus* + *Lactobacillus bulgaricus*; BSM, *Lactiplantibacillus* + *Pediococcus pentosaceus* + *Lentilactobacillus buchneri*; CELL, *Trichoderma* cellulase, SDA, sodium diacetate

The bacterial community composition in fresh alfalfa and alfalfa silage at the phylum level is shown in Fig. 4A. Before ensiling, *Actinobacteriota* were found to be the mainstream phyla, with a relative abundance of 46.65%, followed by *Proteobacteria* (33.94%) in fresh alfalfa. After 60 days of ensilage, the microbial community structure underwent an obvious shift, and the most dominant phyla transformed into *Firmicutes*, accounting for more than 99% in the SK, TS, KL and SDA groups. It’s declared that low pH is beneficial for the growth of *Firmicutes* (Jung et al. 2024; Zhao et al. 2021b). So this may be a contribution to a rapid decline and lower in pH. In contrast, *Actinobacteriota* and *Proteobacteria* was significantly reduced after ensiling, and *Proteobacteria* had a higher relative abundance in the C, LP, and CELL groups. Analogously, studies proclaimed that *Firmicutes* dominated silages regardless of LAB inoculation, whereas the abundance of *Proteobacteria* markedly declined after ensiling (Sun et al. 2023).

Our results displayed that at the genus level, *Nocardioides*, *Pseudomonas*, *Massilia*, and *Sphingomonas* were primary components of the bacterial community in fresh alfalfa, with 11.02%, 8.73%, 7.52%, and 5.26% (Fig. 4B), respectively, but none of them were expected to be genera for good fermentation (Guo et al. 2023). Similarly, Ren et al. (2020) pointed out that there was a parallel community composition in fresh cauliflower leaves, with dominance of *Pseudomonas* and *Massilia*. Moreover, Zhao et al. (2021b) reported that the epiphytic bacterial community of fresh alfalfa was led by genera *Enterobacter* and *Pseudomonas*, but dominant genera were *Enterococcus, Pantoea,* and *Cyanobacteria* in fresh alfalfa (Yang et al. 2019). Only 0.38% *Pediococcus* and no *Lactiplantibacillus* were found in fresh alfalfa, implying an extremely limited number of LAB. Besides, similar results have been reported by Si et al. (2023) and Wang et al. (2022) previously. After 60 days of ensiling, the dominant genera initially present in fresh alfalfa were almost completely replaced by *Lactiplantibacillus,* which turned into the predominant genus in all alfalfa silages except SK (Fig. 4B). Considering the updated taxonomy of *lactobacilli*, *Lactiplantibacillus* was distinguished from *Lactobacillus* in the present study. Therefore, the dominance of *Lactiplantibacillus* observed in the present study likely reflects the strong proliferation of *L. plantarum*-related taxa during alfalfa ensiling. Except for the SK group, the relative abundance of *Lactiplantibacillus* in all groups displayed a significant lift than the control, among which the abundance of *Lactiplantibacillus* was the highest in KL (78.23%), followed by SDA (69.14%), and LP (65.76%), which is in keeping with the higher lactic acid concentration and lower pH and NH_3_-N concentration. Previous studies noted that *L. plantarum* inoculation increased the abundance of *Lactiplantibacillus* (Ren et al. 2020; Zhao et al. 2021b). Results manifested that *L. acidophilus* and *L. bulgaricus* in KL did not alter the dominance of *L. plantarum*. Additionally, it’s speculated that the antibacterial effect of SDA provided an environment conducive to LAB growth, allowing *Lactiplantibacillus* to passively gain dominance, thus forming a structure similar to LP and KL. Both TS and CELL exerted similar driving influences on the microbial community in alfalfa silage. The addition of *P. pentosaceus* led TS group toward a community where fermentation was jointly dominated by *Lactiplantibacillus* and *Pediococcus*. While CELL, although lacking exogenous LAB, may have promoted cell wall degradation through cellulase, releasing more fermentable sugars, thereby enhancing the proliferative dominance of *Lactiplantibacillus* and *Pediococcus*. In our study, SK revealed the highest abundance of *Pediococcus* (61.90%), while the abundance of *Lactiplantibacillus* decreased. It may be ascribed to inclusion of *E. faecium* that forms a synergistic competitive relationship with *P. pentosaceus*, thus reinforcing *Pediococcus*'s early dominance. Furthermore, the early utilization of available substrates by *P. pentosaceus* and *E. faecium* may have limited the substrates available for *L. plantarum*, which could partly explain its reduced dominance during the later stage of ensiling. It should be noted that 16S rRNA amplicon sequencing cannot distinguish the inoculated strains and the indigenous strains of the same species, particularly because the sequencing depth could only resolve to the genus level. Therefore, although LAB-related genera increased in the inoculated silages, it is unclear whether inoculated strains actually persisted until day 60, requiring further investigation through metagenomic sequencing.

There was a significantly lower abundance for *Enterococcus, Serratia,* and *Lactococcus* in the additive-treated alfalfa silage (Fig. 4B). Our result is parallel to the results reported by Jung et al. (2024) and Yang et al. (2019). Although *Enterococcus* is bacteria that initiate early fermentation, their number and activity are significantly reduced once the pH is below 4.5, so they are not generally found in high-quality silage (Yang et al. 2019). This observation corresponds well with the lower pH values detected in the additive-treated silages. In contrast, the higher abundance of *Enterococcus* in the control group may be associated with a higher pH (5.29). The relative abundance of *Enterococcus* in alfalfa silage was reported to exceed 65% at a pH of 5.83 (Ni et al. 2017). The genus *Serratia*, members of *Enterobacteriaceae*, have no ability to ferment WSC to produce lactic acid, but decompose proteins, produce ammonia and biogenic amines, resulting in an increase in NH₃-N during ensiling (Muck 2010). This is consistent with a significantly higher NH_3_-N level in the control group.

### LEfSe analysis of differentially enriched microbial taxa among treatments

**Fig. 5:**
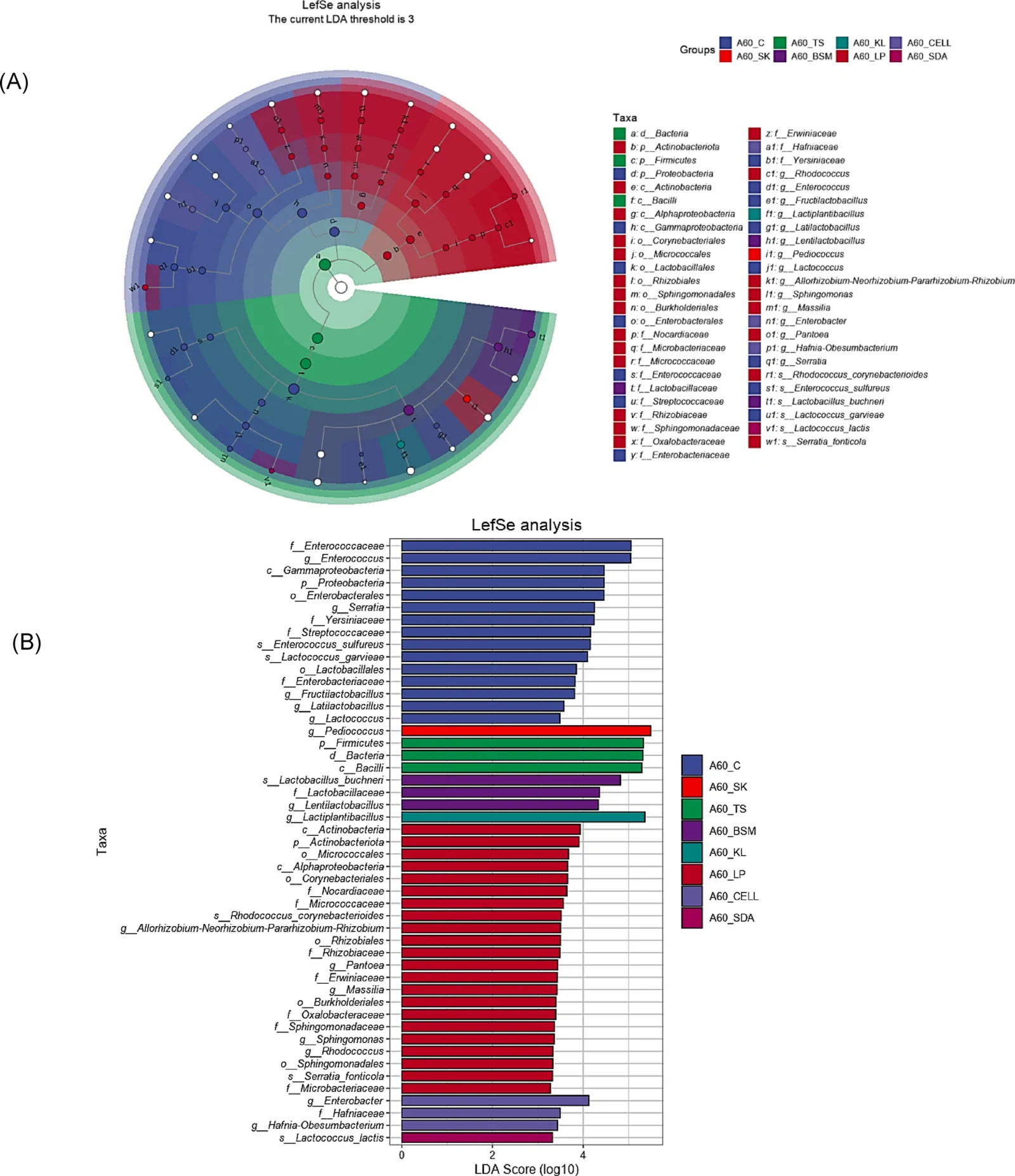
LEfSe analysis of bacterial communities in alfalfa silage treated with different additives after 60 days of ensiling. (A) LEfSe taxonomic cladogram showing the phylogenetic distribution of differentially abundant bacterial taxa among treatments. (B) LDA score plot showing the discriminative bacterial taxa identified by LEfSe analysis. A0, fresh alfalfa; A60, alfalfa silage after 60 days of ensiling; C, control: no additives; LP, *Lactiplantibacillus plantarum*; TS, *Lactiplantibacillus plantarum* + *Pediococcus pentosaceus*; SK, *Lactiplantibacillus plantarum* + *Pediococcus pentosaceus* + *Enterococcus faecium*; KL, *Lactiplantibacillus plantarum* + *Lactobacillus acidophilus* + *Lactobacillus bulgaricus*; BSM, *Lactiplantibacillus* + *Pediococcus pentosaceus* + *Lentilactobacillus buchneri*; CELL, *Trichoderma* cellulase, SDA, sodium diacetate

LEfSe analysis was used to explore whether additive treatments shifted the enrichment in microbial taxa at different levels and set the LDA score threshold to 3 and *p* < 0.05 in the experiment (Fig. 5). The taxonomic cladogram shows that the enriched taxa were distributed across different taxonomic levels (Fig. 5A). In the LDA score plot, each taxon was therefore discussed according to its corresponding taxonomic level (Fig. 5B). At the species level, the taxon labelled as *L. buchneri* was enriched in the BSM group, with LDA being 4.89 (*p* < 0.05), suggesting that the introduction of *L. buchneri* may alter the microbial community compared to TS. *L. buchneri,* a heterofermentative LAB, generates lactic acid and acetic acid (Okoye et al. 2023). It is consistent with the higher AA concentration in the BSM groups. Additionally, *Lactococcus lactis* was significantly enriched in the SDA group at the species level (*p* < 0.05), suggesting that the treatment favored a more LAB-driven fermentation pattern. As a typical homofermentative LAB, *L. lactis* can utilize WSC and convert them mainly into lactic acid through glycolysis, thereby accelerating acidification during ensiling (Okoye et al. 2023; Soundharrajan et al. 2025), which is consistent with the higher lactic acid concentration, lower pH and NH_3_-N concentration observed in the SDA group. At the genus level, *Pediococcus*, *Lactiplantibacillus*, and *Lentilactobacillus* were enriched in the SK, KL, and BSM groups, respectively, and their LDA scores were recorded as 5.48, 5.37, and 3.35 (*p* < 0.05). The enrichment of *Pediococcus* in the SK group may be related to the presence of *P. pentosaceus* in this commercial inoculant. *Pediococcus* and *Lactiplantibacillus* are all homo-fermentative LAB, which can efficiently facilitate WSC to produce lactic acid and acidify the environment to inhibit undesirable bacteria (Okoye et al. 2023), which is in keeping with the lower pH value, NH_3_-N, alpha diversity, and higher lactic acid concentration in these groups. In this study, the *Hafniaceae* family and its subordinate *Enterobacter* and *Hafnia–Obesumbacterium* genera formed the enriched groups in the CELL group (*p* < 0.05), among which *Enterobacter* had the highest LDA of 4.09 (*p* < 0.05). These taxa compete with LAB for fermentable substrates and contribute to proteolysis and the production of alkaline metabolites (Muck 2010), which may explain the relatively higher NH_3_-N concentration in the CELL group.

### Functional pathways in alfalfa silage treated with different additives

**Fig. 6:**
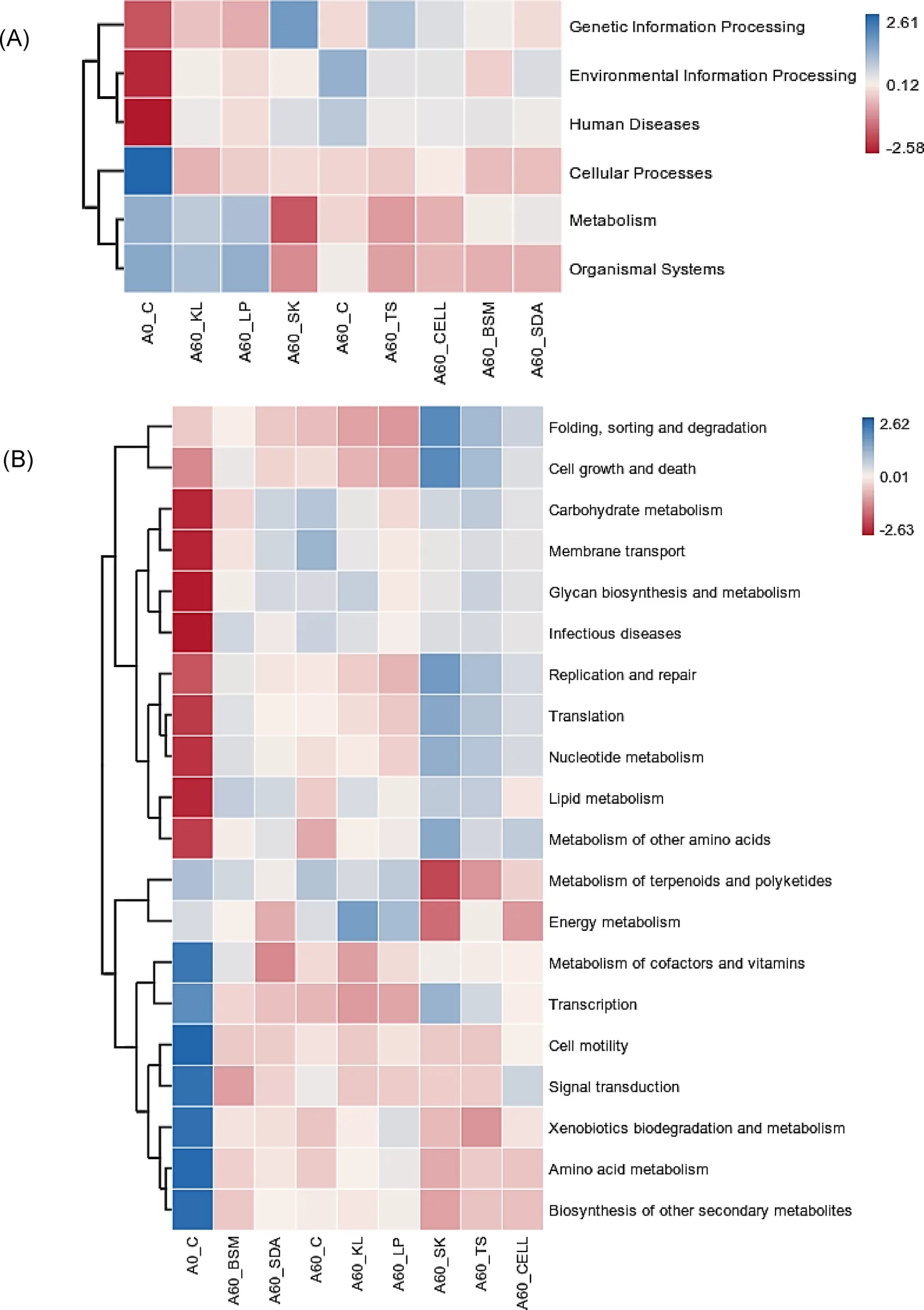

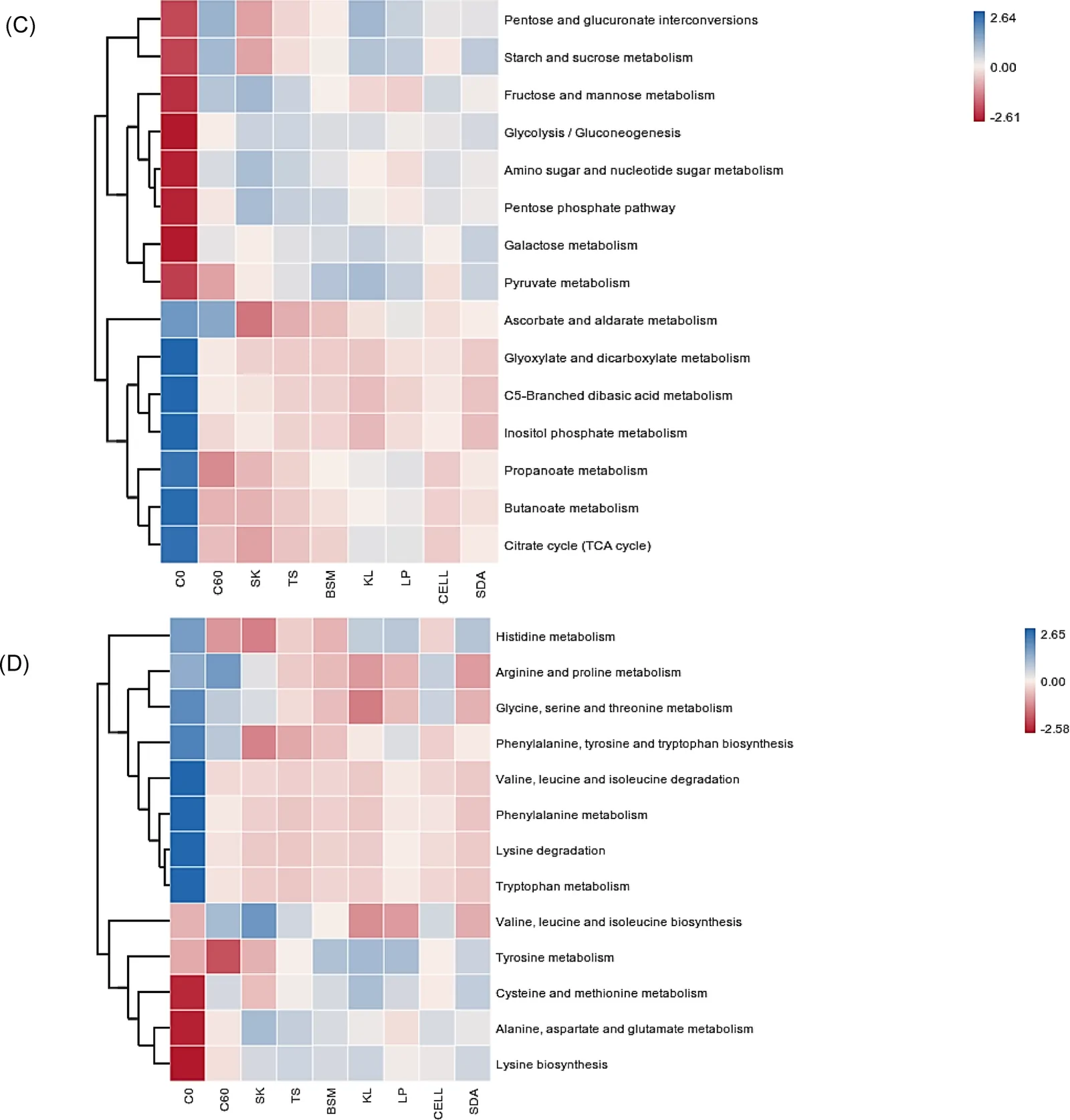
PICRUSt2-predicted functional profiles of bacterial communities in fresh alfalfa and alfalfa silage treated with different additives after 60 days of ensiling. (A) Predicted KEGG level 1 functional categories. (B) Predicted KEGG level 2 functional pathways. (C) Predicted carbohydrate metabolism-related pathways at KEGG level 3. (D) Predicted amino acid metabolism-related pathways at KEGG level 3. A0, fresh alfalfa; A60, alfalfa silage after 60 days of ensiling; C, control: no additives; LP, *Lactiplantibacillus plantarum*; TS, *Lactiplantibacillus plantarum* + *Pediococcus pentosaceus*; SK, *Lactiplantibacillus plantarum* + *Pediococcus pentosaceus* + *Enterococcus faecium*; KL, *Lactiplantibacillus plantarum* + *Lactobacillus acidophilus* + *Lactobacillus bulgaricus*; BSM, *Lactiplantibacillus* + *Pediococcus pentosaceus* + *Lentilactobacillus buchneri*; CELL, *Trichoderma* cellulase, SDA, sodium diacetate

PICRUSt2 was adopted to PICRUSt2 was used to predict the potential functional profiles of bacterial communities in alfalfa silage. These predicted functions were used to provide supplementary information on possible functional shifts associated with changes in bacterial community composition during ensiling. Metabolism was the most abundant pathway, greater than 75% of the predicted functions in all groups, followed by genetic information processing (Fig. 6A). This is in keeping with the study of Wang et al. (2022). LP and KL showed the highest value in predicted metabolism function, while genetic information processing showed the lowest. This pattern may be associated with the dominance of *Lactiplantibacillus* and the relatively favorable fermentation characteristics observed in LP and KL, including lower pH and higher lactic acid accumulation. Conversely, SK had the lowest proportion of predicted metabolism-related functions and the highest genetic information processing, suggesting that the addition of *E. faecium* may be related with intense community competition, a fragmented fermentation pathway, and increased cellular stress and biosynthetic pressure.

At KEGG level 2, Carbohydrate metabolism was marked as the highest abundance in the bacterial predicted functional pathways, followed by predicted amino acid metabolism (Fig. 6B), which is closely related to silage fermentation because carbohydrate metabolism is involved in the utilization of WSC for organic acid production, whereas amino acid metabolism may be associated with protein degradation and nitrogen transformation during ensiling (Bai et al. 2021). At KEGG level 3, several carbohydrate metabolism-related pathways, including glycolysis, pyruvate metabolism, amino sugar and nucleotide sugar metabolism, fructose and mannose metabolism, and the pentose phosphate pathway, showed relatively high predicted enrichment after ensiling (Fig. 6C). This pattern may be associated with LAB enrichment and lactic acid accumulation under an anaerobic environment (Okoye et al. 2023). SK displayed the highest predicted proportions of nucleotide metabolism, replication and repair, transcription, and folding-sorting-degradation, whereas energy and amino acid metabolism were the lowest. This may be attributed to that the addition of *P. pentosaceus* or *E. faecium* increased community competition and stress, leading to higher replication pressure, DNA damage repair needs, and protein folding pressure on cells. Conversely, there were the highest predicted proportions of energy metabolism and amino acid metabolism in KL and LP, while exhibiting the lowest levels of predicted genetic information processing functions. This may be associated with the dominance of *Lactiplantibacillus* and the favorable fermentation characteristics observed in these groups, including higher lactic acid concentration and lower pH. Amino acid decarboxylation, malate decarboxylation, and arginine deamination are three major energy metabolism pathways in LAB, which lead to lactic acid accumulation during fermentation (Pessione et al. 2010). Nevertheless, inoculation with *L. plantarum* + *P. pentosaceus* + *E. faecium* resulted in lower levels than the control group. Previous studies have also proclaimed that inoculation with *P. pentosaceus* or *E. faecium* reduces predicted energy metabolism (Bai et al. 2021). Additives enhanced the predicted abundance of lysine biosynthesis while markedly suppressing the predicted abundance of lysine degradation, phenylalanine metabolism, and tryptophan metabolism, with the most pronounced in KL and SDA (Fig. 6D), likely driven by the lower pH. Du et al. (2025) declared that the reduction in predicted amino acid metabolic pathways may be associated with reduced protein degradation. This is consistent with higher crude protein, lower NH_3_–N concentrations, and lower abundance of *Enterobacteriaceae* in this study.

### Predicted carbon- and nitrogen-related ecological functional potentials based on FAPROTAX analysis

**Fig. 7:**
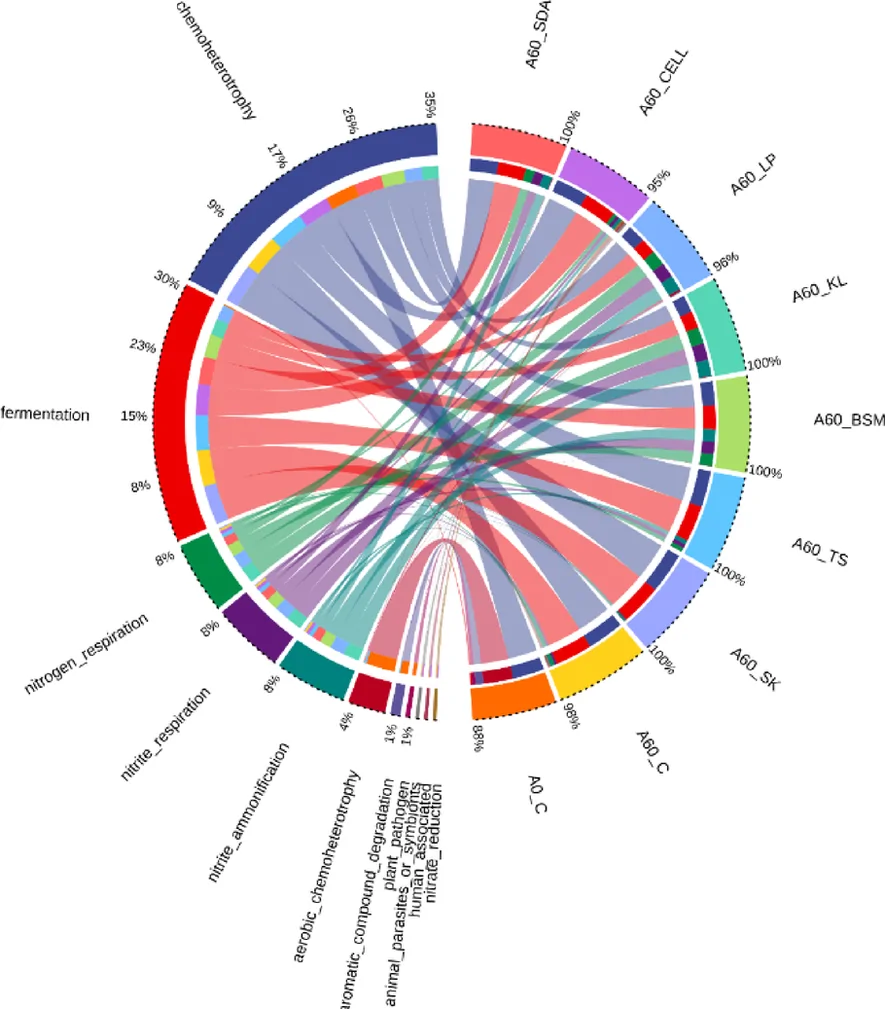
FAPROTAX-based prediction of ecological functional potentials of bacterial communities in fresh alfalfa and alfalfa silage treated with different additives after 60 days of ensiling. A0, fresh alfalfa; A60, alfalfa silage after 60 days of ensiling; C, control: no additives; LP, *Lactiplantibacillus plantarum*; TS, *Lactiplantibacillus plantarum* + *Pediococcus pentosaceus*; SK, *Lactiplantibacillus plantarum* + *Pediococcus pentosaceus* + *Enterococcus faecium*; KL, *Lactiplantibacillus plantarum* + *Lactobacillus acidophilus* + *Lactobacillus bulgaricus*; BSM, *Lactiplantibacillus* + *Pediococcus pentosaceus* + *Lentilactobacillus buchneri*; CELL, *Trichoderma* cellulase, SDA, sodium diacetate

In the present study, FAPROTAX results were regarded as exploratory predictions of ecological functional potentials and were used only as supplementary information to support the interpretation of bacterial community shifts. The abundance of predicted fermentation was significantly increased after ensiling, whereas the control group showed a higher predicted fermentation and chemoheterotrophy abundance compared to all groups except SK (Fig. 7). This may be related to the higher bacterial diversity and higher pH in the control silage. It has been reported that the genera *Enterobacter, Klebsiella, Serratia*, and *Escherichia* have strong NH_3_- generating ability (Zhang et al. 2019). There was a lower abundance in nitrogen-related predicted functions, including nitrogen respiration, nitrite respiration, and nitrite ammonification, but a higher NH_3_-N concentration. Thus, this suggests that NH₃-N accumulation may be more closely associated with protein degradation and deamination by undesirable bacteria. KL and LP reported relatively higher abundances of nitrogen-related predicted functions. Simultaneously, considering the rapid pH decline and lower NH_3_-N concentration, and high *Lactiplantibacillus* abundance, these phenomena may indicate potential changes in the nitrogen-related functional metabolism of bacterial communities under faster acidification. An interesting phenomenon was that the abundance of predicted nitrogen respiration, nitrite respiration, and nitrite ammonification functions decreased with increasing *P. pentosaceus* abundance, except for the control group, in our experiment. These observations suggest that *P. pentosaceus* may be associated with shifts in predicted nitrogen-related functions, including nitrogen respiration, nitrite respiration, and nitrite ammonification in alfalfa silage. However, since FAPROTAX predictions are based on taxonomic inference rather than direct functional measurements, these results should be interpreted as inferred functional trends. Further metagenomics or transcriptomic are needed to clarify whether *P. pentosaceus* directly influences nitrogen transformation pathways.

## Conclusions

This study showed that all additives effectively improved fermentation quality by modulating the abundance of *Lactiplantibacillus* and *Pediococcus*, regulating predicted metabolic pathways and reducing undesirable bacteria. Among microbial inoculants, KL appeared to be the most effective, as reflected by the lowest gas loss and NH₃–N content, and simplified bacterial community, along with the highest *Lactiplantibacillus* abundance, while BSM was less effective in this study. SDA displayed the lowest pH, gas loss and NH₃–N concentration, and higher lactic acid concentration and *Lactiplantibacillus* abundance, while cellulase treatment was less effective than microbial inoculants and SDA. Summarily, KL and SDA displayed the most outstanding performance in reducing gas loss, pH, NH₃–N, and a more simplified LAB-dominated microbial community. These findings highlight that different additives regulate fermentation through distinct microbiota-mediated mechanisms and provide practical guidance for optimizing alfalfa silage production. Nevertheless, this study was performed at a controlled bag-silage scale, and larger-scale trials under practical farm conditions are needed to validate the applicability of these findings.

## Abbreviations

ADF: Acid Detergent Fiber
ADL: Acid Detergent Lignin
ASV: Amplicon Sequence Variant
BSM: Commercial Microbial Inoculant Containing *Lactiplantibacillus plantarum* + *Pediococcus pentosaceus* + *Lentilactobacillus buchneri*
C: Control
CELL: Cellulase
CFU: Colony-Forming Unit
CP: Crude Protein
DM: Dry Matter
DMR: Dry Matter Recovery
FAPROTAX: Functional Annotation of Prokaryotic Taxa
FM: Fresh Matter
GL: Gas Loss
KEGG: Kyoto Encyclopedia of Genes and Genomes
KL: Commercial Microbial Inoculant Containing *Lactiplantibacillus plantarum* + *Lactobacillus acidophilus* + *Lactobacillus*. *bulgaricus*
LAB: Lactic Acid Bacteria
LDA: Linear Discriminant Analysis
LEfSe: Linear Discriminant Analysis Effect Size
*L. acidophilus*: *Lactobacillus acidophilus*
*L. buchneri*: *Lentilactobacillus buchneri*
*L. bulgaricus*: *Lactobacillus*. *bulgaricus*
*L. lactis*: *Lactococcus lactis*
*L. plantarum*: *Lactiplantibacillus plantarum*
LP: Commercial Microbial Inoculant Containing *Lactiplantibacillus plantarum*
NDF: Neutral Detergent Fiber
NH₃–N: Ammonia–Nitrogen
PCoA: Principal Coordinate Analysis
*P. pentosaceus*: *Pediococcus pentosaceus*
PERMANOVA: Permutational Multivariate Analysis of Variance
PICRUSt2: Phylogenetic Investigation of Communities by Reconstruction of Unobserved States 2
QIIME2: Quantitative Insights Into Microbial Ecology 2
SDA: Sodium Diacetate
SK: Commercial Microbial Inoculant Containing *Lactiplantibacillus plantarum* + *Pediococcus pentosaceus* + *Enterococcus faecium*
TN: Total Nitrogen
TS: Commercial Microbial Inoculant Containing *Lactiplantibacillus plantarum* + *Pediococcus pentosaceus*
*E. faecium*: *Enterococcus faecium*
WSC: Water-Soluble Carbohydrate
